# Obesogenic behaviors during structured periods among children and adolescents with intellectual and developmental disabilities: a systematic review and meta-analysis

**DOI:** 10.1186/s12966-026-01881-5

**Published:** 2026-02-05

**Authors:** Keagan P. Kiely, Keith Brazendale, McKenna Hill, Sarah Burkart, Michael W. Beets, Elizabeth L. Adams, Bridget Armstrong, Christine St. Laurent, Abigail Hogan, James W. White III, Olivia Finnegan, Joshua Culverhouse, Anthony Holmes, R. Glenn Weaver

**Affiliations:** 1 Department of Exercise Science, Arnold School of Public Health, University of South Carolina, 921 Assembly Street, Columbia, South Carolina 29208 USA; 2 Department of Health Sciences, University of Central Florida, Orlando, Florida USA; 3 Department of Kinesiology, University of Massachusetts Amherst, Amherst, Massachusetts USA; 4 Department of Communication Sciences and Disorders, University of South Carolina, Columbia, South Carolina USA

**Keywords:** Obesogenic behaviors, Intellectual disabilities, Developmental disabilities

## Abstract

**Background:**

Children and adolescents with intellectual and developmental disabilities (IDD) are at greater risk for obesity and poor obesogenic behaviors (e.g., physical activity, screen time, diet, sleep) than their typically developing counterparts. The Structured Days Hypothesis (SDH) suggests that in typically developing children and adolescents, obesogenic behaviors worsen during periods of reduced structure (e.g., weekend or summer vacation). However, children and adolescents with IDD have unique factors that may alter how structure (i.e., pre-planned, segmented, adult supervised, out-of-home programs) influences obesogenic behaviors. Therefore, the objective of this systematic review and meta-analysis is to examine obesogenic behaviors during periods of more and less structure among children and adolescents with IDD.

**Methods:**

A comprehensive search of PubMed, PsycINFO, Embase, and Web of Science was performed through the end of 2024 based on the PICO framework. Studies were eligible if they included youth with IDD and measured obesogenic behaviors across contexts with differing degrees of structure. Two reviewers independently completed the screening process, extracted all relevant information, and evaluated methodological quality using the NHLBI tool. Results were synthesized using fixed- and random-effects meta-analyses and visually represented with forest plots.

**Results:**

A total of 4,236 papers were screened with 323 full-text articles retrieved. After screening, 33 total studies were identified (physical activity = 23, sedentary behaviors = 12, sleep = 11, diet = 1). Meta-analyses indicated that the standardized mean difference of physical activity (Random = 0.27, [95%CI: 0.13–0.40], *p* < 0.00), and diet (0.16, [95%CI: 0.03–0.29], *p* = 0.02) aligned with the SDH while sleep (Random = -0.01, [95%CI: -0.16-0.14], *p* = 0.88), sedentary and screen time (Random = -0.01, [95%CI: -0.38-0.36], *p* = 0.95) did not align.

**Conclusions:**

Periods of greater structure were associated with more favorable physical activity and diet outcomes among children and adolescents with IDD, although evidence for dietary behaviors was limited. Findings support the relevance of the SDH in this population while highlighting substantial gaps in the literature, including small study numbers and methodological heterogeneity. Future research using rigorous, longitudinal designs is needed to better understand the relationship between structure and obesogenic behaviors among children and adolescents with IDD.

**Supplementary Information:**

The online version contains supplementary material available at 10.1186/s12966-026-01881-5.

## Background

The Structured Days Hypothesis (SDH) posits that structure may be effective at promoting healthy behaviors in typically developing children. SDH defines structure as a pre-planned, segmented, adult-supervised, and compulsory environment and posits that structure functions as a protective agent against accelerated BMI gains and worsening obesogenic behaviors [1]. Evidence has shown that typically developing children gain more weight during the three months of summer than they do during the entire 9 months of school [2–5]. When structure is removed, such as during the summer, evidence shows increased sedentary time [6, 7], reduced physical activity (PA) [7–11], later and inconsistent sleep times [12–14], and nutrient-poor, energy-dense diet [15–21]. Consistent with these behavioral changes, separate studies have reported accelerated BMI gains during the summer months. Over 150 peer-reviewed studies support the SDH, demonstrating consistent findings that obesogenic behaviors worsen during period of less structure among typically developing children [1].

Children with intellectual and developmental disabilities (IDD) are characterized by life-long deficits in intellectual and adaptive functioning which have direct impact on maintaining healthy body mass index (BMI), leading to them being twice as likely to experience obesity compared to typically developing children [22–24]. IDD consists of etiologically specific conditions like Down syndrome or Fragile X syndrome or functional classifications such as impairments in intellectual, physical, or adaptive functioning. While the extent and nature of the deficit of each child varies across the IDD spectrum, these impairments often lead to distinct behavioral patterns, greater reliance on caregivers, unequal access to daily structure, and participation in programs that differ in availability and design compared to typically developing peers. The effects of these characteristics often result in worse obesogenic behaviors (e.g., lower physical activity, poorer diets, greater screen time, disrupted sleep), compared to typically developing children [25–30].

Obesity may be more complex in children and adolescents with IDD due to unique characteristics related to their diagnosis. For example, etiological-specific conditions like Prader-Willi and Down syndrome are associated with endocrine abnormalities and low fat-free mass, causing the maintenance of a healthy BMI to be more difficult [31, 32]. Additionally, impaired adaptive behaviors (e.g., communication, self-care, or conceptual skills) or sensory issues in other IDD may influence participation in prompted activities or lead to narrow diets due to food aversions. Conversely, if individuals with IDD have difference that cannot be altered, they may benefit more from downstream effects of a structured environment. For example, structure (e.g., school) may provide calorically capped meals, periods of physical activity with appropriate adaptations, reduced access to screens, and an anchor in which families can implement healthy rules and routines around (e.g., bedtimes). These unique characteristics attributed to IDD illustrate distinct influences that may alter how the SDH framework functions in children and adolescents with IDD and highlight the need to examine the SDH in this specific population.

There has been no systematic review of literature that examines obesogenic behaviors during periods of structure in children and adolescents with IDD. The SDH, in its current form, was not designed to describe the relationship between structure and obesogenic behaviors in children and adolescents with IDD. Due to the reasons outlined above, it is unclear if these periods of reduced structure impact obesogenic behaviors in the same manner. Thus, the purpose of this paper is to review and meta-analyze the current body of literature examining the relationship between periods of structure (e.g., school year or weekdays) and obesogenic behaviors in children and adolescents with IDD.

## Methods

This systematic review and meta-analysis was conducted and reported in accordance with the PRISMA 2020 guidelines and the checklist is in Supplemental Table 6 [33].

### Search strategy

PubMed, PsycINFO, Embase, and Web of Science databases were used to identify relevant articles published before December 31st, 2024, no additional studies were identified after this date. The search strategy used the PICO framework, aligning search components with what is the population (P); the intervention/exposure (I); comparison (C), and outcome (O) [34]. Our search terms for the four PICO categories are as follows: (P) Youth with IDD (e.g., children and adolescents with intellectual and/or developmental disabilities), (I) Structure (e.g., weekday, school), (C) Less/more structure (e.g., weekend, summer), and (O) Behavior (e.g., physical activity, screen time, sleep, diet). Terms were truncated and combined using Boolean operators. An example of key term/MeSH terms is provided in supplementary Table 1. Additional manual searches were conducted using reference lists of previous reviews to identify any additional articles.

### Eligibility criteria

**Fig. 1:**
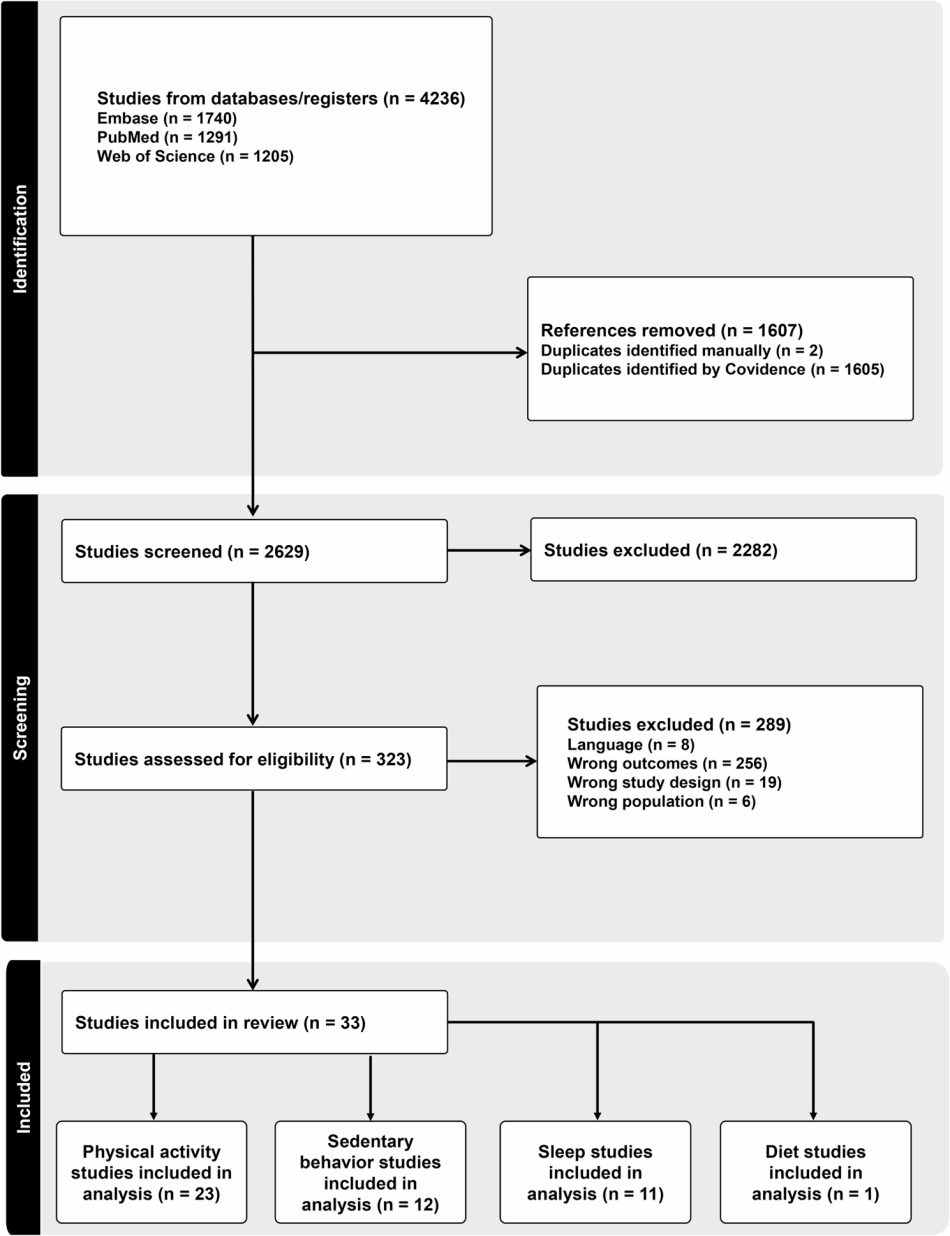
PRISMA flow chart

To be eligible for inclusion, studies met the following criteria: (i) observational design or report baseline data from an intervention trial. Observational studies were eligible because the paper sought to examine behaviors void of any intentional changes from an intervention. Interventional study designs were not excluded because, in theory, the baseline of intervention is analogous to a cross-sectional design; (ii) include participants who were children (aged 5–12 years) and/or adolescents (aged 12 to 18 years), or report a mean participant age that fell within these age ranges; (iii) participants diagnosed with at least one intellectual or developmental disability (defined in introduction); (iv) provide point estimates and variance of at least one obesogenic behavior across two timepoints with different amounts of structure (e.g., school vs. summer/vacation, weekday vs. weekend); and (v) published in the English language. Any studies that compared time within the same day (e.g., classroom versus recess) or reported time in a behavior as a proportion were excluded. Poster or conference presentations and dissertation work were not included. There were no geographical restrictions. The PRISMA flow diagram outlining the screening for eligible papers is pictured in Fig. 1.

### Identification of relevant studies

All studies returned by the searches in each database were uploaded to Covidence Systematic Review Management Program, where duplicates were automatically removed (*n* = 1,607). Two authors (KK and MH) independently examined all potential studies by title and abstract to ensure they did not have any clear exclusion criteria (e.g., examined behaviors in only adults) before reaching a full-text screening. All articles that met the inclusion criteria after full-text screening were passed on to the extraction phase. In instances of disagreement concerning study inclusion, the authors (KK and MH) met to discuss the uncertainty and made a final decision together.

### Data extraction

Key descriptors and outcomes measures were extracted from all eligible studies. Descriptors consisted of citation details (first author, year of publication), country, sample size, number and proportion of female participants, study region, and disability related information (e.g., diagnosis or disability inclusion criteria), type of comparison of structure (e.g., weekend vs. weekday or School vs. Summer), and outcome measured along with the measurement tool and procedures (e.g., accelerometer, self-report, PA thresholds). Sedentary behaviors consisted of both sedentary time and screen time. Extraction for screen time consisted of all day screen time and screen time after 8pm. PA consisted of moderate-to-vigorous PA (MVPA), energy expenditure, total PA, and steps. Extraction for sleep consisted of sleep duration (e.g., total sleep time), sleep onset, and sleep offset. Diet just consisted of a quantity of specified food categories (e.g., dairy, fast food, fruit, salty snacks, sugar sweetened beverages (SSB), sweets, and vegetables). For studies that reported median and interquartile range and/or range, an equation was used to transform them into mean and standard deviation [35]. When information was missing (e.g., sample size of subgroup in analysis) an attempt was made to contact the corresponding author to retrieve missing information before using the available data or eliminating the study from the analysis.

### Risk of bias assessment

The risk of bias for each study was assessed using the NHLBI Study Quality Assessment Tool for Observational Cohort and Cross-Sectional Studies [36]. This assessment consists of 14 questions that two separate raters (KK and MH) answer to assess the potential flaws in study methods that may lead to sources of bias. Each question could be answered with either met, did not meet, did not report, or not applicable. Any discrepancies regarding the risk of bias were resolved through discussion between the two reviewers (KK and MH), who reached a consensus decision. Risk of bias was used descriptively and not as an exclusion criterion or as a sensitivity analysis due to the limited number of available studies.

### Data analysis

The standardized difference in means (SDM) and its standard error was calculated for each outcome within a study using Comprehensive Meta-Analysis (CMA, Version 4; Biostat, Englewood, NJ). The SDMs were coded in a manner that all positive effects supported the SDH (e.g., increased MVPA during periods with more structure) while negative effects did not support the SDH (e.g., decreased MVPA during periods with more structure). To synthesize findings across studies, we estimated pooled effects using both fixed- and random-effects models. The fixed-effects model assumes that all included studies estimate a common underlying effect size and that observed differences between studies arise solely from sampling error. In contrast, the random-effects model incorporates between-study variability by assuming that each study estimates a related true effect size. This approach accounts for heterogeneity attributable to differences in study populations, measurement protocols, or contextual factors. For this reason, random-effects models were considered primary due to expected heterogeneity across populations, diagnoses, and measurement tools. Presenting results from both models allows us to evaluate the robustness of the pooled estimates under varying assumptions about heterogeneity and provides a more comprehensive interpretation of the synthesized evidence. Effect sizes of ≤ 0.20 were considered small, effects from > 0.20–0.79 were considered medium, and effect sizes ≥ 0.80 were considered large [37]. Based on the guidance from the Cochrane Handbook for Systematic Reviews of Interventions we estimated and presented both fixed and random effects models [35]. The I² statistic was used to assess heterogeneity among effect estimates for each outcome. Thresholds of ≤ 30%, > 30%-60%, ≥ 60% were interpreted as low, moderate, and high heterogeneity, respectively [35]. A one-study-removed sensitivity analysis was employed to assess the influence of individual studies and identify potential outliers. Potential moderators were included in the Comprehensive Meta-Analysis data set, and a meta-regression was used to evaluate the influence of these potential moderators. Age was coded as children (5–12 years old), adolescent (≥ 13 years old), or both if the same included both age ranges. Diagnosis was coded, autism spectrum disorder, cerebral palsy, developmental coordination disorder, intellectual disability, Prader-Willi syndrome, and Down syndrome. Measurement tool was either objective, subjective, or both if the study used both (e.g., parent-reported sleep and accelerometry). Relevant information was exported to excel and forest plots were created for visual representation of effect sizes and 95% confidence intervals.

## Results

A combined total of 4,236 studies were identified from PubMed, Web of Science, and Embase and uploaded to Covidence. Covidence then removed 1,605 duplicates with authors removing two more leaving a total of 2,629 titles and abstracts to be screened for relevance. A total of 2,282 studies were deemed irrelevant, and 323 studies moved to full-text screening. Of these 323 studies 292 studies were excluded due to various exclusion criteria. This left a total of 33 studies for data extraction.

Table 1 describes all studies that were included in the analysis of this paper. Of these studies, PA was the obesogenic behavior represented across most studies (*n* = 23), followed by sedentary time (*n* = 8), sleep (*n* = 11), screen time (*n* = 5) and diet (*n* = 1). A total of 11 studies measured more than one behavior and only a single study measured all four behaviors. Most studies (*n* = 30) examined differences between weekdays and weekend days, two examined school and summer differences, and one study examined days children participated in summer day camps versus summer days they did not attend. Cross-sectional study design was the most common study design (*n* = 32), only one was intervention study in which the baseline data was used. Twenty-three studies used an objective measure of obesogenic behaviors. Among all studies, 14 studies aligned with the SDH, 5 did not align, and the remaining 14 studies were mixed (e.g., measured multiple behaviors and some behaviors aligned and some did not).

**Table 1:** Table of included studies

Author, Year	Sample Size^a^/Female	Ages Included ^b^	Disability	Country	Study Design	Structure Comparison	Objective Measure	Behaviors Measured	Sleep Outcomes Measured	Screen Time Outcomes Measured	Physical Activity Outcomes Measured	Diet Outcomes Measured	Align/Not Align^c^
Akyurek, 2024 [38]	150/53	Children	Mixed	Turkey	Cross-sectional	Weekend	No	Screen Time	-	Total	-	-	Not Align
Alhusaini, 2018 [27]	37/0	Children	DS	Saudi Arabia	Cross-sectional	Weekend	Yes	Physical Activity	-	-	Steps	-	Not Align
Allik, 2006 [39]	32/4	Children	ASD	Sweden	Cross-sectional	Weekend	Both^d^	Sleep	Onset, Offset, Duration	-	-	-	Mixed
Allik, 2008 [40]	16/2	Adolescent	ASD	Sweden	Cross-sectional	Weekend	Yes	Sleep	Onset, Offset, Duration	-	-	-	Align
Brazendale, 2021 [41]	17/7	Children	Mixed	United States	Cross-sectional	SDC	Both^e^	Physical Activity, Diet, Sleep, Screen Time	Onset, Offset, Duration	Total, Screen time after 8pm	MVPA, Steps	% of days consumed	Mixed
Brazendale, 2023 [42]	14/4	Children	ASD	United States	Cross-sectional	Summer	No	Sleep, Screen Time	Onset, Offset, Duration	Total, Screen time after 8pm	-	-	Mixed
Brazendale, 2024 [43]	6/2	Children	ASD	United States	Cross-sectional	Summer	Yes	Physical Activity, Sedentary Time, Sleep	Onset, Offset, Duration	-	Sedentary, MVPA	-	Align
Castner, 2014 [28]	24/12	Both	PW	United States	Cross-sectional	Weekend	Yes	Physical Activity	-	-	MVPA	-	Align
Einarsson, 2015 [25]	91/29	Both	ID	Iceland	Cross-sectional	Weekend	Yes	Physical Activity	-	-	Counts, MVPA	-	Align
Fonvig, 2023 [44]	381/157	Both	CP	Denmark	Cross-sectional	Weekend	No	Sleep, Screen Time	Duration	Total	-	-	Mixed
Garcia-Pastor, 2019 [45]	44/11	Both	ASD	Spain	Cross-sectional	Weekend	Yes	Physical Activity, Sedentary Time	-	-	Steps, Sedentary, MVPA	-	Mixed
Hao and Razman, 2022 [46]	275/92	Both	ID	Malaysia	Cross-sectional	Weekend	No	Physical Activity, Sedentary Time	-	-	Sedentary, MVPA	-	Mixed
Helsel, 2024 [47]	123/60	Both	DS	United States	Cross-sectional	Weekend	Yes	Physical Activity	-	-	MVPA	-	Mixed
Inthikoot, 2020 [29]	65/19	Children	ASD	Thailand	Cross-sectional	Weekend	No	Sleep	Onset, Offset, Duration	-	-	-	Mixed
Izquierdo-Gomez, 2014 [48]	90/34	Adolescent	DS	Spain	Cross-sectional	Weekend	Yes	Physical Activity, Sedentary Time	-	-	Sedentary, MVPA	-	Mixed
Kim, 2009 [49]	16/3	Both	Mixed	United States	Cross-sectional	Weekend	Yes	Physical Activity	-	-	Steps, Counts	-	Align
Memari, 2013 [50]	80/35	Both	ASD	United States	Cross-sectional	Weekend	Yes	Physical Activity	-	-	Counts	-	Mixed
Mkrtchyan, 2022 [51]	32/NR	Children	ID	Armenia	Intervention (Baseline-only)	Weekend	Yes	Physical Activity	-	-	Steps	-	Not Align
Must, 2014 [30]	53/9	Children	ASD	United States	Cross-sectional	Weekend	No	Screen Time	-	Total	-	-	Align
Nicholson, 2017 [52]	116/35	Both	CP	United States	Cross-sectional	Weekend	Yes	Physical Activity	-	-	Steps	-	Mixed
Pan, 2011 [53]	35/NR	Children	ASD	Taiwan	Cross-sectional	Weekend	Yes	Physical Activity	-	-	Counts, MVPA	-	Mixed
Pan, 2016 [54]	35/0	Both	ASD	Taiwan	Cross-sectional	Weekend	Yes	Physical Activity	-	-	Counts, MVPA	-	Align
Pan, 2021 [55]	68/0	Both	ASD	Taiwan	Cross-sectional	Weekend	Yes	Physical Activity, Sedentary Time	-	-	Counts, MVPA, Sedentary	-	Mixed
Queralt, 2016 [56]	35/13	Both	Mixed	Spain	Cross-sectional	Weekend	Yes	Physical Activity	-	-	Steps	-	Align
Smit, 2020 [57]	36/14	Both	CP	Netherlands	Cross-sectional	Weekend	Yes	Sleep, Physical Activity, Sedentary Time	Duration	-	Active Time, Sedentary	-	Mixed
Sung, 2023 [58]	68/0	Both	ASD	Taiwan	Cross-sectional	Weekend	Yes	Physical Activity, Sedentary Time	-	-	Counts	-	Align
ven Wely, 2012 [59]	62/23	Both	CP	Netherlands	Cross-sectional	Weekend	Yes	Physical Activity	-	-	Steps	-	Align
van Rijssen, 2023 [60]	38/18	Children	CP	Netherlands	Cross-sectional	Weekend	Yes	Sleep	Duration	-	-	-	Not Align
Wachob, 2015 [61]	10/1	Both	ASD	United States	Cross-sectional	Weekend	Yes	Physical Activity	-	-	MVPA	-	Align
Wiggs, 2016 [62]	30/5	Both	DCD	United Kingdom	Cross-sectional	Weekend	Yes	Sleep	Onset, Offset, Duration	-	-	-	Align
Wright, 2006 [63]	178/79	Both	Physical Disability	Canada	Cross-sectional	Weekend	No	Sleep	Duration	-	-	-	Not Align
Yang, 2024 [64]	32/15	Both	DS	South Korea	Cross-sectional	Weekend	Yes	Physical Activity, Sedentary Time	-	-	Steps, MVPA	-	Align
Yuan, 2022 [65]	346/172	Both	ID	China	Cross-sectional	Weekend	No	Physical Activity	-	-	Energy Expenditure, MVPA	-	Align

*Abbreviations: SDC* Summer Day Camp, *ASD* Autism Spectrum Disorder, *CP* Cerebral Palsy, *DS* Down Syndrome, *DCD* Developmental Coordination Disorder, *ID* Intellectual Disability, *PW* Prader-Willi Syndrome, *MVPA* Moderate-to-Vigorous Physical Activity

^a^ The size of the sample used for comparison

^b^ Children = 6–12 years old, Adolescent = 13+, Spans both early and late

^c^ Align = All measured behaviors improved on more structured days, Not Align = All measured behaviors worsened on more structured days, Both=Some of the measured behaviors improved and some worsened on more structured days

^d^ Allik, 2006 used both objective and survey measures to measure sleep

^e^ Screen time and diet were measured using a survey, PA and sleep were measured using accelerometry

**Fig. 2:**
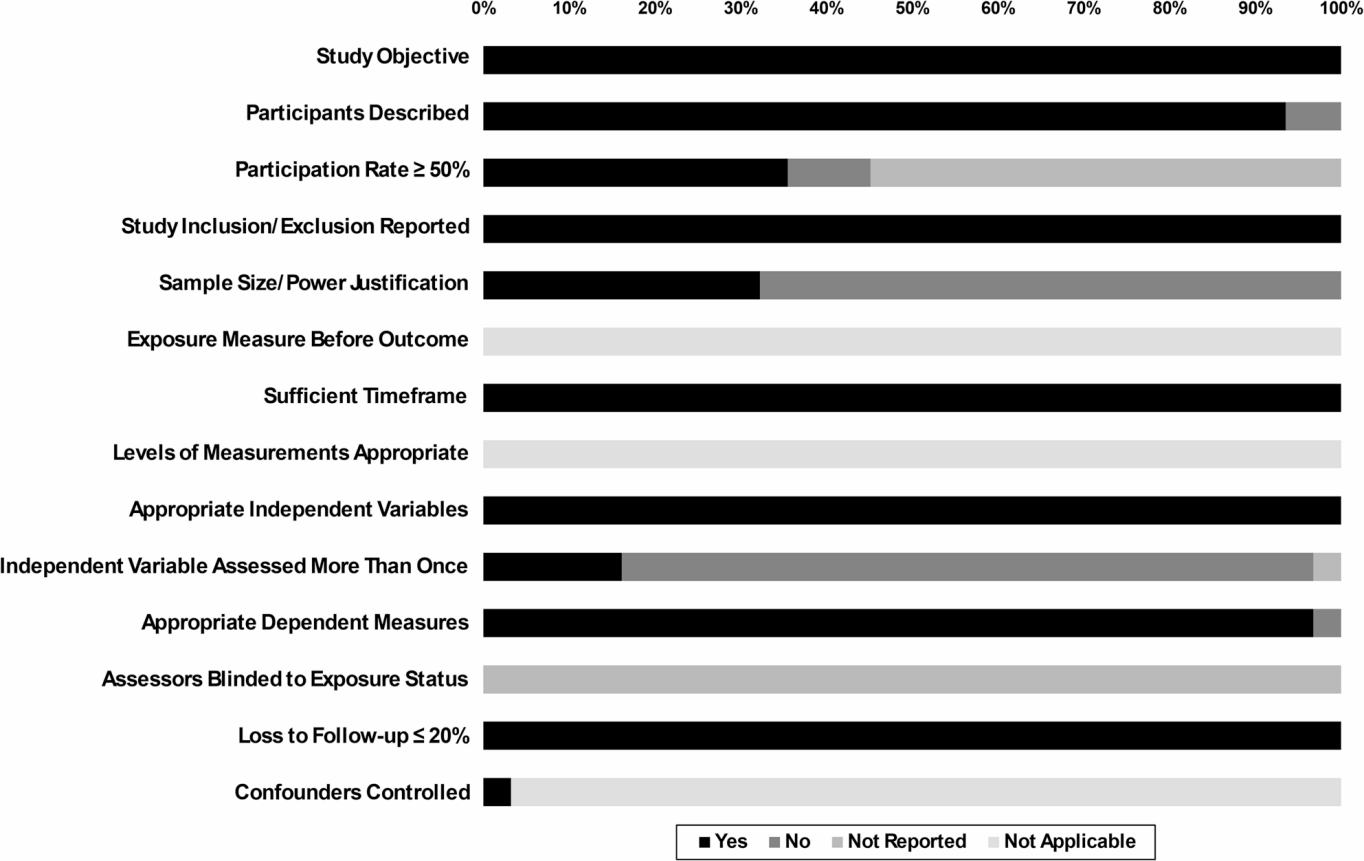
Risk of bias among included studies

The findings from the risk of bias assessment are presented in Fig. 2. All studies articulated explicit objectives (100%) and most provided sufficient descriptions of their participant samples (94%). The majority also clearly defined independent (100%) and dependent (97%) variables. Furthermore, only 16% of studies implemented repeated measurements of the independent variable (e.g., measured two weeks of weekdays and weekends) Among studies, 35% achieved participation rates of at least 50%, however 55% of studies did not report participation rates (e.g., percentage of individuals from the target population who were contacted and eligible to participate in a study). Additional inclusion and exclusion criteria were specified in 100% of studies, while sample size justification was reported in 32%. Blinding procedures were not reported by any studies and 100% of studies had less than 20% attrition. A detailed breakdown of the results for each paper is shown in Supplemental Table 5.

**Fig. 3:**
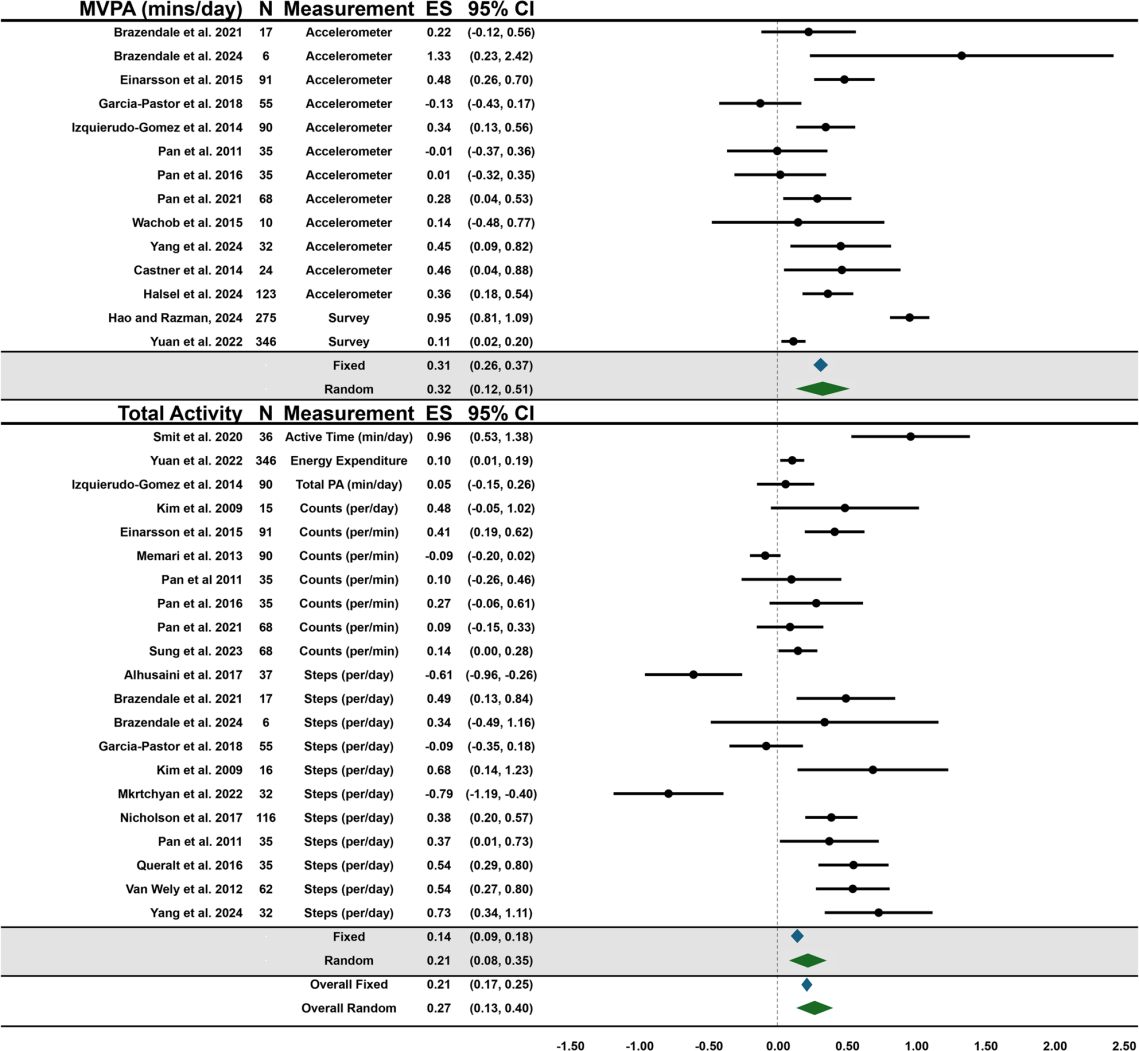
Forest plot of summary effects for PA outcomes

**Fig. 4:**
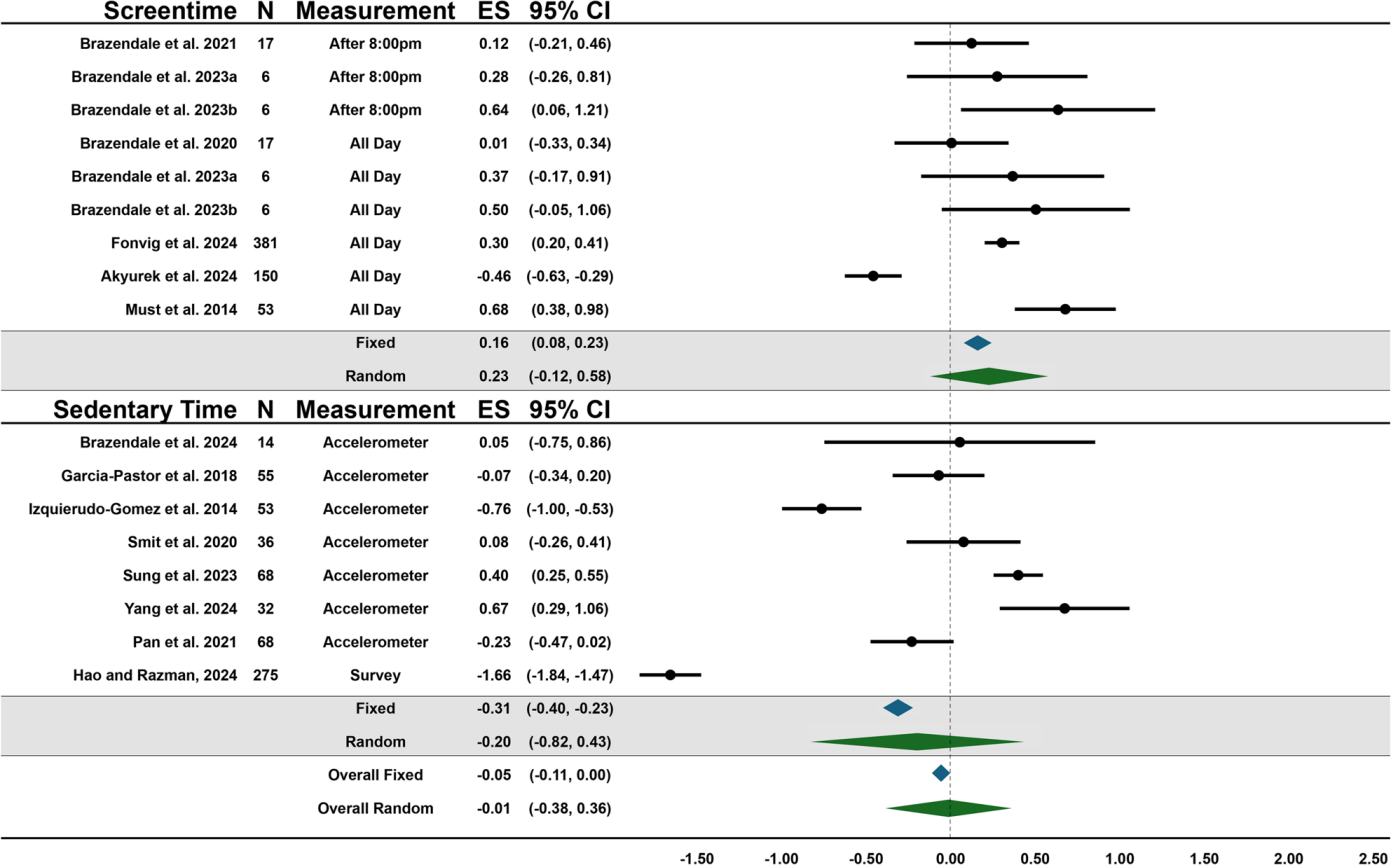
Forest plot of summary effects for sedentary behavior outcomes. ^a^ Brazendale et al. 2023 [42] data from school to summer ^b^ Brazendale et al. 2023 [42] data from high/low structure during the summer

**Fig. 5:**
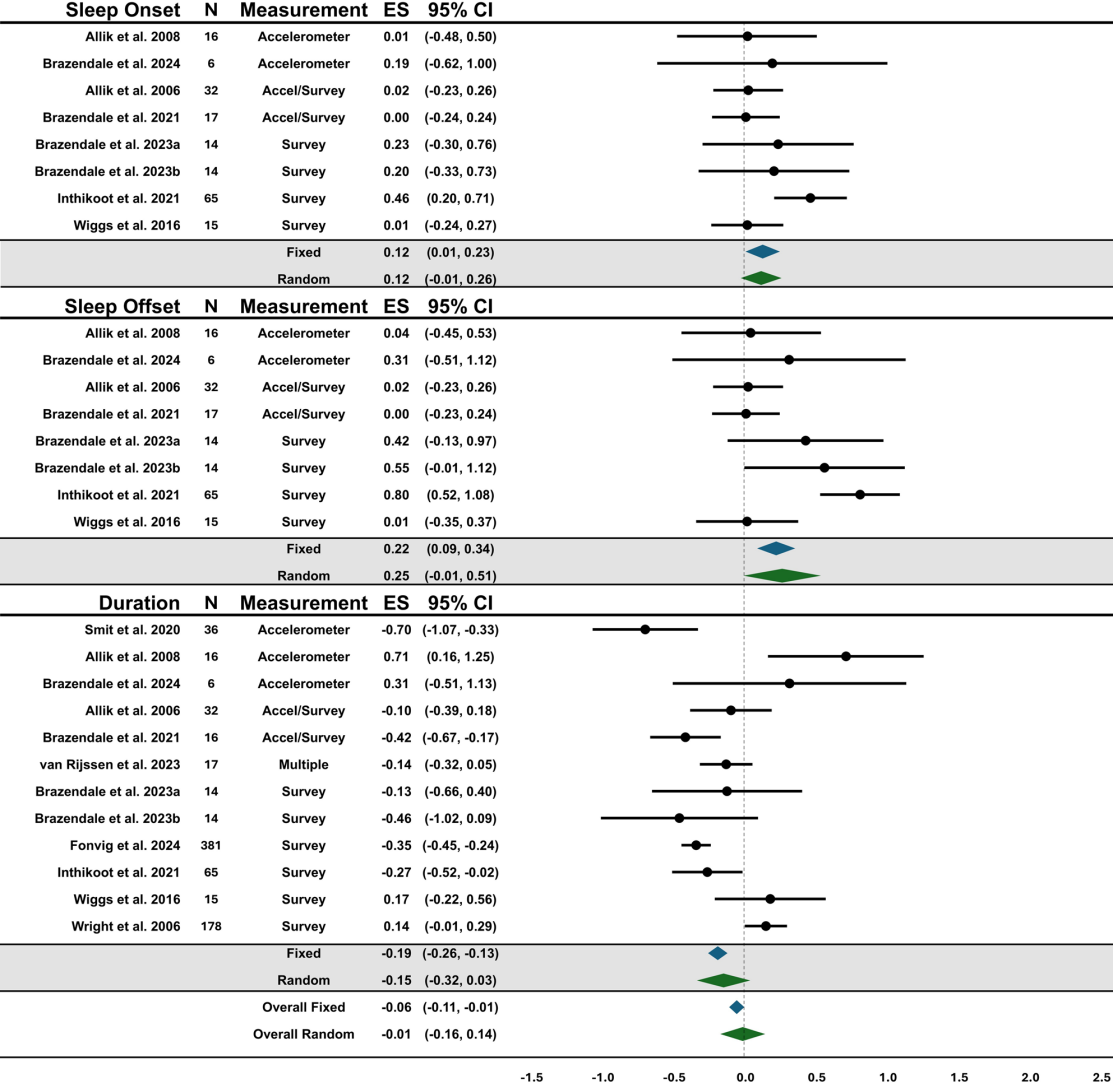
Forest plot of summary effects for sleep outcomes. ^a^ Brazendale et al. 2023 [42] data from school to summer ^b^ Brazendale et al. 2023 [42] data from high/low structure during the summer

**Fig. 6:**
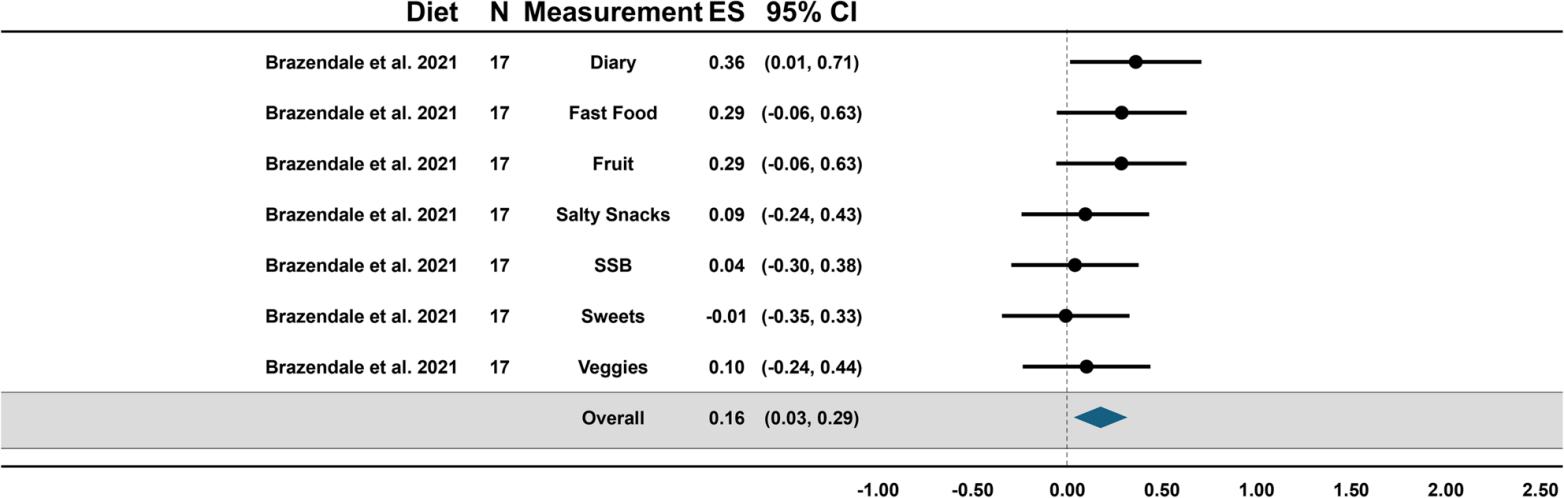
Forest plot of summary effects for diet

Forest plots showing the overall summary fixed and random effects for behaviors and the effects of each outcome within behaviors are presented in Figs. 3, 4, 5 and 6. Due to the large heterogeneity (e.g., I^2^) across fixed effect models, the results of the random effects were used for conclusions; however, the fixed effects are reported for context and transparency.

### Physical activity

There was a moderate overall effect for PA (Random = 0.27, [95%CI: 0.13–0.40], *p* < 0.00; Fixed = 0.21, [95%CI: 0.17–0.25], *p* < 0.00) indicating PA was higher during periods with more structure. There was a high level of heterogeneity within the fixed-effects model (I^2^ = 92.19%) and medium in the random-effects model (I^2^ = 31.75%). MVPA effect size was positive and statistically significant with a moderate effect (Random = 0.32, [95%CI: 0.12–0.51], *p* < 0.00; Fixed = 0.31, [95%CI: 0.26–0.37], *p* < 0.00). Total activity (steps, counts, energy expenditure, etc.) also supported the SDH and was statistically significant with a small effect (Random = 0.21, [95%CI: 0.08–0.35], *p* < 0.00; Fixed = 0.14, [95%CI: 0.09–0.19], *p* < 0.00). Meta-regression analyses indicated that diagnosis, age, or measurement tool was not significantly associated with the pooled effect size. Supplemental Table 2. shows the effect sizes from the one-study-removed analysis which ranged from 0.16 to 0.27 (Fixed) and 0.24–0.29 (Random).

### Sedentary & screen time

The overall effect for sedentary behaviors (sedentary and screen time) was small and did not support the SDH (Random = -0.01, [95%CI: -0.38-0.36], *p* = 0.95; Fixed = -0.05, [95%CI: -0.11-0.00], *p* = 0.06). There was a high level of heterogeneity within the fixed-effects model (I^2^ = 97.55%) and no heterogeneity within the random-effects model (I^2^ = 0.0%). The indication of no heterogeneity by the I^2^ in the random effects model is likely due to the low number of studies and how the model estimates variance. The overall effects for sedentary time did not support the SDH (Random = -0.20, [95%CI: -0.82-0.43], *p* = -0.53; Fixed = -0.31, [95%CI: -0.40- -0.23], *p* < 0.00) demonstrating no difference in sedentary time during periods of more structure compared to periods of less structure. Screen time had a small-moderate effect size (Random = 0.23, [95%CI: -0.12-0.58], *p* = 0.19; Fixed = 0.16, [95%CI: 0.08–0.23], *p* < 0.00) and showed that amount of screen time did not differ during periods of more structure compared to periods with less. Meta-regression analyses indicated that diagnosis was significantly associated with the pooled effect size (β = 0.3, 95% CI [0.04–0.56], *p* = 0.02), suggesting that studies examining only children and adolescents with ASD were more likely to report less sedentary behaviors during periods with more structure, compared to other IDD. Age and measurement tool was not associated with the pooled effect size. Supplemental Table 3 shows the effect sizes from the one-study-removed analysis which ranged from − 0.07 to 0.14 (Fixed) and − 0.05–0.15 (Random).

### Sleep

The overall effect for sleep was small-moderate but was not statistically significant (Random = -0.01, [95%CI: -0.16-0.14], *p* = 0.88; Fixed = -0.06, [95%CI: -0.11- -0.01], *p* = 0.03). There was a high level of heterogeneity within the fixed-effects model (I^2^ = 86.42%) and small in the random-effects model (I^2^ = 5.24%). A moderate effect was seen for total sleep duration and did not support the SDH (Random = -0.15, [95%CI: -0.32-0.03], *p* = 0.10; Fixed = -0.19, [95%CI: -0.26- -0.13], *p* < 0.00) suggesting sleep duration was shorter during periods with more structure. Sleep onset (Random = 0.12, [95%CI: -0.01-0.26], *p* = 0.08; Fixed = 0.12, [95%CI: 0.01–0.23], *p* = 0.04) and sleep offset (Random = 0.25, [95%CI: -0.01-0.51], *p* = 0.06; Fixed = 0.22, [95%CI: 0.09–0.34], *p* < 0.00) were not statistically significant. However, the directionality of the effects shows sleep onset and offset were later during periods with less structure. Meta-regression analyses indicated that diagnosis was significantly associated with the pooled effect size (β = -0.33, 95% CI [-0.49- -0.16], *p* < 0.01), suggesting that studies examining only children and adolescents with cerebral palsy were more likely to report worse sleep during periods of more structure, compared to other IDD. Supplemental Table 4. shows the effect sizes from the one-study-removed analysis which ranged from − 0.05 − 0.03.

### Diet

While there was only one study that examined diet, it included two separate comparisons (e.g., summer camp weekdays vs. no summer camp weekdays and summer camp weekdays vs. summer weekend days) so we ran a single fixed model to examine the overall effects. The effects of diet were small and supported the SDH (0.16, [95%CI: 0.03–0.29], *p* = 0.02). All measures of diet were positive except consumption of sweets but not statistically significant, and dairy was the only statistically significant effect observed (0.36, [95%CI: 0.01–0.71], *p* = 0.04). A meta-regression and one-study-removed were not run for this study because there was only a single study analyzed.

## Discussion

This systematic review meta-analyzed studies examining obesogenic behaviors across periods with more or less structure (e.g., weekdays versus weekends, school versus summer) in children and adolescents with IDD. Overall, the meta-analysis of 33 studies showed 14 studies reporting better obesogenic behaviors during periods of more structure, as defined by the SDH, compared to less-structured times (e.g., summer and weekend days), while 14 reported mixed results, and 5 did not align with the SDH.

Measures of PA (e.g., MVPA, steps, counts per minute) had the largest pooled effect across studies, signifying that children and adolescents with IDD had greater levels of PA during periods of more structure. Approximately 82% of the studies examining PA had positive effects (i.e., increases during periods with more structure). This finding mirrors research in typically developing children, wherein 80% of studies reviewed showed an increase in PA during periods with more structure (1). Within measures of PA, MVPA had the largest effect and was statistically significant. Across the 14 studies that measured MVPA, the SDM ranged from 0.31 (fixed-effects) to 0.32 (random-effects) which equates to approximately 13.5 min/per day more MVPA during periods with more structure. Similarly, measures of total PA (e.g., steps, counts per minute) showed consistent positive effects, wherein 17 of 21 studies showed increases in PA during periods with more structure. The results of the one-study-removed analysis suggested that no single study disproportionately influenced the overall results, reinforcing the robustness of the findings that children and adolescents with IDD improve PA during periods with more structure. While promising that structure may be related to increased PA, the average MVPA across all studies was still below the recommended 60 min per/day [66]. Structured programs may not be enough to adequately reach the recommended amount of PA, therefore additional strategies to provide more supportive environments within the structured programs (e.g., proper adaptation physical education class and the inclusive recess within the school setting and afterschool programing) may help increase PA levels to a greater extent [67, 68].

Within studies examining screen time, all except one study supported the SDH (e.g., reduced screen time during periods with more structure). These findings align with structure being a protective agent, if a child is in structure where screens are inaccessible, then the amount of time that can be filled with screen use is greatly reduced [69]. However, screen time use in this population is nuanced and there may be an underlying need for screens (e.g., communication) that may not be present for typically developing children. For example, these individuals may require a device with a screen for augmentative and alternative communication or to provide equitable learning experience. Further, this population may benefit from exergame that utilize virtual reality or game-based learning on a computer or tablet. These examples highlight a key distinction between compulsory use for school and therapy versus leisure screen use. However, the current literature examining difference in screen time between periods of more or less structure does not make a distinction between these types of screen use [30, 38, 41, 42, 44]. Instead, the studies included rely on parent-reported durations of computer, television, and phone use without distinction of purpose. Even without these distinctions, a majority of studies included showed increased screen time on weekends, when compulsory use of screens would likely be reduced [30, 41, 42, 44].

Conversely, sedentary time was not statically different among periods with more structure compared to periods with less structure, however this finding is nuanced. One study in particular showed significantly higher levels of sedentary time on weekdays compared to weekends [46]. This large difference was present across different ages, genders, BMI, and diagnosis severity, with no group achieving less sedentary time on weekdays compared to weekends. This result might stem from their use of a questionnaire-based assessment of sedentary time. The Children’s Leisure Activities Study Survey-Chinese edition (CLASS-C) allows a parent to report the duration and frequency of eight different sedentary activities, one of which is attending class. It is possible that this measure inflates sedentary time simply by reporting the frequency and duration of class time, an activity that would presumably be on the weekday but not be present during the weekend. Further, the broad generalization that time spent in a classroom is sedentary may not be appropriate due to the integration of movement within classrooms as a result of classroom rules and routines [70]. When we conducted a one-study-removed sensitivity analysis the overall effect of sedentary activities shifted to positive (e.g., support the SDH). This indicates that the overall findings may be sensitive to the inclusion of this study and should be interpreted with caution.

Sleep duration was shorter and sleep onset and offset were earlier during periods with more structure. This shift in sleep patterns is consistent with the SDH among typically developing children and adolescents [1]. Increased sleep duration during periods with less structure may be a result of compensating for less sleep during structured periods (e.g., the school week), but the later bed and wake times are associated with increased risk for obesity and worsening obesogenic behaviors in typically developing children and adolescents [71–76]. A study examining the sleep pattern of over 380 adolescents with IDD showed that, after controlling for sleep durations, individuals who were categorized as early bedtime/waketime were less likely to experience obesity and were more physically active than individuals categorized as late bedtime/waketime [77]. Conversely, the effect size associated with sleep onset reflected a shift to ~ 46 min later bedtime, less extreme than that seen in typically developing children (70–90 min) [78–80]. Sleep offset had slightly more variability with children and adolescents waking up approximately ~ 60 min later during periods with less structure. It has been shown in previous literature that consistent routines, like bedtime, are often implemented to help with sleep and behavioral problems [63, 81, 82]. While the findings of this review showed a positive relationship between structure and sleep onset/offset, the effect was moderate and may reflect more consistent bedtimes in this population, regardless of structure. It is important to interpret the results of these findings in the context of other influences, such as family rules and routines.

### Limitations

The shortcomings of this review highlight the gaps in literature examining obesogenic behaviors in children and adolescents with IDD. A notable limitation is not only the small pool of studies but the lack of studies across all behaviors. As stated earlier, PA was the most studied behavior with 23 studies examining various aspects of PA; however, there were only 5 studies examining screen time. Relying on a small collection of studies impacts the ability to generalize the pooled estimate from these studies, an issue made more complex by the variability in study characteristics among these studies (e.g., measurement tool, sample size, disability). The findings of this paper may offer early evidence that the SDH framework may function similar in children and adolescents with IDD, yet more research is needed to form a well-supported conclusion.

The research on school versus summer is very limited and the only study that used objective measures from school to summer only had 6 participants. Further research is needed evaluating the difference in these obesogenic behaviors in the school year compared to summer. When examining weekend behaviors, it is logical to presume a caregiver would, to some extent, be able to keep routines, activities, and overall structure of the day consistent for these 48 h. However, when days with less structure are extended, like during summer break, caregivers may be limited in their ability to keep similar routines and behaviors consistent due to constraints in resources (e.g., time of work, money for day camps, availability of developmentally appropriate camps). This could have a two-fold negative impact, worsening of obesogenic behaviors of the child and/or increased burden on caregiver to provide structure that is unable to be captured during the weekend. While examining seasonal differences in BMI gains and obesogenic behaviors in children with IDD, these studies could explore secondary outcomes related to household stress and other indirect outcomes that may be negatively impacted during the summer.

Second, only two studies examined sleep, physical activity, and sedentary behaviors, together, allowing for a more granular view of changes in these behaviors in the context of a 24-hour framework. For example, Brazendale et al. (2023) showed that children with autism spectrum disorder had slightly more sleep on school days which resulted in a shift of less sedentary time and light PA and more MVPA compared to summer days [80]. Similarly, Smit et al. (2020) measured sleep duration, sedentary time, and active time, enabling an examination of how time allocated to these behaviors differed between weekdays and weekends [83]. While it is important to understand how structure impacts obesogenic behaviors, a more complete approach to understanding structure’s impact on these behaviors is to examine how time is allocated across these three behavior categories. For instance, a day in which sleep duration is shortened and replaced with more sedentary time is less favorable than a day in which that time is filled with some form of PA. Building upon this 24-hour movement behaviors perspective, future research can use time-use methodology to identify where and with whom these individuals accumulate increases in PA versus sedentary time. Ultimately, allowing researchers to integrate time-use methodology with 24-hour movement behavior measures to examine the context (e.g., where and with whom) that optimal behaviors occur to inform future interventions targeting obesogenic behaviors in children and adolescents with IDD [84].

### Implications

This review provides important implications for the prevention and treatment of obesity in children and adolescents with IDD. The SDH has been instrumental in our understanding of the etiology of obesity and our ability to treat and prevent it in typically developing children. The extension of this framework to children and adolescents with IDD could provide additional paths of addressing obesogenic behaviors and improving BMI in this population that mirror those of typically developing children. Similar to the literature of typically developing children, MVPA is significantly improved during periods with more structure, suggesting that interventions intended to improve PA in this population may want to target periods of reduced structure, opposed to adaptive current forms of structure. Conversely, diet and sleep intervention may have more difficulty materializing meaningful outcomes in this population as our review shows these behaviors varied minimally across varying amounts of structure. Demonstrating that structure can improve behaviors and health outcomes in children and adolescents with IDD may have additional implications for clinical practices. Structure may act as a low-level but persistent exposure that works in conjunction to ongoing interventional therapies. Clinicians may also benefit from monitoring seasonal periods of increased risk and modify current standard of care and provide contextual recommendations. The findings of this paper suggest that extending the SDH to children and adolescents with IDD may provide clinically relevant guidance for monitoring periods of increased health risk and integrating structure as a complementary component of standard care.

### Future directions

Future research should aim to address the weaknesses addressed previously while continuing to build the pool of literature examining obesogenic behaviors during periods of structure among children and adolescents with IDD. Future research should prioritize larger sample sizes with consistent methodologies, examining multiple obesogenic behaviors across extended periods of reduced structure, such as the summer. Further, it is important that research begins examining the relationship between changes in obesogenic behaviors and changes in BMI and begins to identify population specific factors (e.g., individual- and family-level) that may explain the variation in outcomes, allowing for better generalizability and mechanistic understanding. As this line of research grows and our understanding deepens, structure’s benefits may be used to inform decision-making efforts of practitioners and policy development.

## Conclusions

This paper is the first to show that the SDH may function similar in children and adolescents with IDD as it does in typically developing children. The results of this paper present the possibility that obesogenic behaviors improve during periods with more structure in children and adolescents with IDD. However, the limited number of studies included and the overall heterogeneity of findings in this paper require caution when interpreting. More studies are needed to better understand how obesogenic behaviors are influenced by structure in this population. Future studies should aim to improve our understanding of school to summer differences by examining obesogenic behaviors over extended unstructured periods like summer. Additionally, future studies can examine obesogenic behaviors together within a 24-hour movement behavior framework to better understand highlighting the need for time-use and contextual analysis to identify when, where, and with whom optimal behaviors occur. Such integrated approaches could guide interventions to prevent obesity and promote health in this high-risk population.

## Supplementary Information

Supplementary Material 1.

## Data Availability

The datasets used and/or analyzed during the current study are available from the corresponding author on reasonable request.

## Abbreviations

IDD: Intellectual and Developmental Disabilities
SDH: Structured Days Hypothesis
PA: Physical Activity
BMI: Body Mass Index
MVPA: Moderate-to-Vigorous Physical Activity
SSB: Sugar Sweetened Beverages
SDM: Standardized Difference in Means
CLASS-C: Children’s Leisure Activities Study Survey-Chinese edition
